# DLX2 promotes gastric cancer epithelial– mesenchymal transition and malignant progression through the PI3K/AKT signaling pathway

**DOI:** 10.3389/fonc.2025.1669890

**Published:** 2025-10-30

**Authors:** Wenjing Chen, Xietao Chen, Xuanfu Chi, Wenpiao Yu, Jinji Jin, Jun Cheng

**Affiliations:** ^1^ Department of General Surgery, First Affiliated Hospital, Wenzhou Medical University, Wenzhou, Zhejiang, China; ^2^ Alberta Institute, Wenzhou Medical University, Wenzhou, Zhejiang, China

**Keywords:** gastric cancer, DLX2, EMT, PI3K/AKT, therapeutic target

## Abstract

**Introduction:**

Gastric cancer (GC) is a major health challenge globally, with poor outcomes often due to late-stage diagnosis and aggressive tumor behavior. This study examines the role of DLX2 in GC progression, focusing on its activation of the PI3K/AKT pathway and induction of EMT, which promote tumor cell proliferation, migration, and anchorage-independent growth. We hypothesize that DLX2 is an independent prognostic marker and modulates the tumor immune microenvironment.

**Methods:**

TCGA RNA sequencing data was analyzed to assess DLX2 as a prognostic factor. In vitro experiments with cell transfection and Western blotting confirmed the effects of DLX2 on EMT and the PI3K/AKT pathway. Functional assays and in vivo models evaluated the impact of DLX2 on tumor cell migration, invasion, and growth. Immune scoring analysis explored the relationship between DLX2 and the tumor immune microenvironment.

**Results:**

High DLX2 expression correlated with reduced survival rates. In vitro and in vivo studies showed that DLX2 overexpression enhanced EMT, activated the PI3K/AKT pathway, and increased tumor cell migration and invasion. Immune scoring analysis indicated a significant association between DLX2 expression and immune/stromal scores.

**Discussion:**

DLX2 emerges as a key regulator in GC malignancy and a potential therapeutic target. Its association with the tumor immune microenvironment suggests a role in GC treatment. Future research should explore DLX2-targeted therapies to enhance GC patient outcomes, offering a promising direction for precision oncology.

## Introduction

Gastric cancer (GC) is one of the most common malignant tumors worldwide, ranking fifth in incidence and fourth in mortality globally. In 2022, over 968,000 new cases of GC were diagnosed, with nearly 660,000 related deaths reported (1). Although the survival rate of patients with GC has improved in several countries, particularly South Korea, Japan, and parts of Europe (2), GC remains one of the most lethal malignancies, with a 1-year survival rate among the lowest and a 5-year survival rate of approximately 30% (3). This poor prognosis is largely attributed to delayed diagnosis, high aggressiveness, metastatic potential, and resistance to chemotherapy. Therefore, a deeper understanding of the molecular mechanisms underlying GC progression is crucial for developing more effective targeted therapies.

A key process in tumor development is epithelial–mesenchymal transition (EMT), which plays a pivotal role in tumor cell invasion and metastasis (4–6). Further research on EMT is essential for improving cancer treatment and patient prognosis. EMT is regulated by multiple factors and signaling pathways, including the PI3K/AKT/mTOR pathway (7), GSK-3β phosphorylation signaling (8), the Notch pathway (9), and the TGF-β pathway (10). The phosphatidylinositol 3-kinase (PI3K)/protein kinase B (AKT) signaling pathway is a central regulator of cell growth, proliferation, migration, metabolism, and survival (11, 12). PI3K is a lipid kinase that transmits intracellular signals and regulates various cellular processes (13). It is classified into three categories: classes I, II, and III (14, 15). AKT, a key downstream effector of PI3K signaling, modulates critical processes such as apoptosis inhibition, cell growth, and metabolic regulation (13). Emerging evidence suggests that activation of the PI3K/AKT signaling pathway is closely associated with EMT-related marker expression in various tumor cells. For example, in breast cancer cells, activation of this pathway is linked to decreased E-cadherin expression and increased N-cadherin and vimentin expression (16). However, the upstream regulators governing PI3K/AKT activation in GC, particularly those influencing EMT, remain largely uncharacterized.

Distal-less homeobox 2 (DLX2) is a member of the DLX gene family and encodes a transcription factor involved in embryonic development and oncogenesis (17). Members of the DLX family exhibit diverse functions in tumors, including DLX4, which is highly expressed in non-small cell lung cancer and promotes cell proliferation and cell cycle progression via the YB-1/CKS2 axis (18), DLX5 activates the NOTCH pathway to enhance invasiveness in osteosarcoma (19), and DLX3 may act as a tumor suppressor in cutaneous squamous cell carcinoma by inhibiting the EGFR-ERBB2 pathway (20).

Studies have demonstrated that DLX2 expression is associated with tumor invasion and metastasis (21). Aberrant DLX2 expression has been reported in a variety of malignancies, including hematologic cancers, breast cancer, and gastric cancer (22–26). Low DLX2 expression negatively correlates with immune activation–related pathways, suggesting that DLX2 may promote tumor immune evasion by suppressing the immune microenvironment (27). In gastric cancer, high DLX2 expression has been detected in tumor tissues but not in adjacent normal tissues, and elevated DLX2 expression is associated with poor prognosis (24). In osteosarcoma, DLX2 has been shown to bind HOXC8 to repress CDH2 transcription, thereby promoting epithelial–mesenchymal transition (EMT). However, whether the unfavorable outcomes mediated by DLX2 in gastric cancer are linked to EMT remains unknown. Notably, the PI3K/AKT signaling pathway is well known to promote tumor metastasis in multiple cancers by regulating EMT (28, 29). Despite these insights, the role of DLX2 in gastric cancer progression—particularly its interaction with EMT and PI3K/AKT signaling—remains largely unexplored.

In this study, we hypothesize that DLX2 promotes GC malignancy by activating the PI3K/AKT pathway to induce EMT, thereby increasing tumor proliferation, invasion, and metastasis. Our objectives were to establish DLX2 as an independent prognostic marker in GC, elucidate its role in modulating the tumor immune microenvironment, and define its mechanistic link with PI3K/AKT activation and EMT. Our findings enhance the current understanding of GC pathogenesis and highlight DLX2 as a promising therapeutic target for precision oncology.

## Materials and methods

### Database bioinformatics analysis

The gastric cancer RNA sequencing transcriptome and clinical data were downloaded from The Cancer Genome Atlas (TCGA) database. The GSE54129 dataset was obtained from the Gene Expression Omnibus (GEO), and the probe matrix was annotated to identify gene symbols. Expression data were then extracted for subsequent analysis. The R limma package was used for difference analysis, and the survival package was used for survival analysis. The ggpubr, vioplot, ggExtra, and ggplot2 packages were used for tumor microenvironment analysis, immune infiltration analysis, drug sensitivity analysis, and immunotherapy analysis.

### Cell lines and culture conditions

Human gastric cancer cell lines (AGS, HGC-27, and MKN-45) and the normal gastric mucosal epithelial cell line GES-1 were obtained from the Cell Bank of the Chinese Academy of Sciences (Shanghai, China). The cells were cultured in RPMI 1640 medium or DMEM (VivaCell Biosciences, China) supplemented with 10% fetal bovine serum (Inner Mongolia Opcel Biotechnology Co., Ltd., China) at 37°C in a humidified atmosphere containing 5% CO_2_.

### Cell transfection

Gastric cancer cells (AGS, HGC-27) in the logarithmic growth phase were seeded into 6-well plates at 70–80% confluency. Transfection was performed via Lipo8000™ transfection reagent (Beyotime, China) according to the manufacturer’s protocol. After 48 h, the transfected cells were harvested for subsequent experiments. For the overexpression of DLX2, we used pcDNA3.1(+) vectors (Invitrogen, USA) containing the full-length human DLX2 cDNA sequence. The exogenous DLX2 was tagged with a Flag epitope at the N-terminus to facilitate detection and purification. The construct was verified by DNA sequencing. For stable expression, the Tet-on system (Clontech, USA) was employed, allowing inducible expression of DLX2 under the control of doxycycline. DLX2-specific siRNA (RiboBio Co., Ltd., Guangzhou, China) was used to knock down endogenous DLX2 expression.

### Western blotting

The cells or tissues were lysed in RIPA buffer (Solarbio, China) containing PMSF. Protein concentrations were determined via a BCA assay kit (Beyotime, China). Proteins were denatured in 5× SDS–PAGE loading buffer (Beyotime) at 100 °C for 10 min, separated by SDS–PAGE, and transferred to PVDF membranes (Solarbio). The membranes were blocked with rapid blocking buffer, incubated with primary antibodies at 4 °C overnight, and then probed with HRP-conjugated secondary antibodies for 1 h at room temperature. The signals were detected via SuperSignal™ West Pico PLUS Chemiluminescent Substrate (Thermo Fisher Scientific).

The antibody used was against E-cadherin (1:1000, Cat# 3195; Cell Signaling Technology). N-cadherin (1:1000, Cat# 13116; Cell Signaling Technology), Snail (1:1000, Cat# 1879; Cell Signaling Technology), PI3K (1:1000, Cat# 4249; Cell Signaling Technology), p-Akt (1:1000, Cat# 4060; Cell Signaling Technology), DLX2 (1:1000, Cat# 26244-1-AP; Proteintech), GAPDH (1:30000, Cat# SA30-01; HUABIO), and HRP-conjugated secondary antibodies (1:20000, Cat# HA1024; HUABIO) were used.

### Cell proliferation assay

Cells (4 × 10³/well) were seeded into 96-well plates. At 24, 48, and 72 h posttransfection, 10 µL of CCK-8 reagent (Beyotime) was added to each well. After 1 h of incubation, the absorbance at 450 nm was measured via a microplate reader (BioTek).

### Wound healing and Transwell assays

Wound Healing: Confluent monolayers in 6-well plates were scratched with a sterile pipette tip. Images were captured at 0, 48, and 72 h via an inverted microscope (Olympus). Migration/Invasion: For the migration assays, 5 × 10^4^ cells in serum-free medium were seeded into Transwell chambers (Corning). For invasion assays, chambers precoated with Matrigel (BD Biosciences) were used. After 8–12 h, the migrated/invaded cells were fixed, stained with 0.1% crystal violet, and counted under a microscope.

### *In vivo* xenograft model

Female BALB/c-nu mice (4–6 weeks old) were purchased from GemPharmatech Co., Ltd. (Jiangsu, China). All animal experiments were approved by the Institutional Animal Care and Use Committee and complied with relevant ethical guidelines. DLX2-overexpressing gastric cancer cells (1×10^6^ cells) were subcutaneously injected into BALB/c-nu mice. Tumor growth was monitored every 2 days. Dox (1 mg/mL) was administered orally to induce DLX2 expression. Tumors were harvested, fixed, and subjected to hematoxylin–eosin (HE) staining and Ki–67 immunohistochemistry. All experimental mice were euthanized via cervical dislocation following deep anesthesia induced by isoflurane inhalation. Anesthesia was administered using 5% isoflurane in 100% oxygen at 1 L/min flow rate for induction (3–5 minutes), maintained at 2-3% isoflurane until loss of consciousness. Death was confirmed by absence of corneal reflex and respiratory arrest for >60 seconds.

### Statistical analysis

The data were analyzed via R 4.1.2, GraphPad Prism 8.0.1, and ImageJ 1.51. The results are presented as the means ± SDs. The results of two groups of data were analyzed via an unpaired t test. *P* < 0.05 indicates a statistically significant difference, except for other explanations (ns *P* > 0.05, * *P* < 0.05, ** *P*<0.01, *** *P* < 0.001, **** *P* < 0.0001).

## Results

### DLX2 as an independent prognostic factor

**Figure 1:**
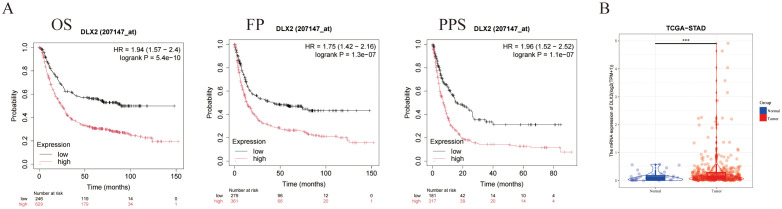
DLX2 expression and its impact on cancer prognosis. **(A)** Kaplan-Meier survival curves illustrating the association between DLX2 expression levels and cancer prognosis: (i) Overall survival (OS) with a hazard ratio (HR) of 1.94 (95% CI: 1.57 - 2.4), log-rank *P* = 5.4e-10; (ii) Disease-free survival (FP) with a HR of 1.75 (95% CI: 1.42 - 2.16), log-rank *P* = 1.3e-07; (iii) Progression-free survival (PPS) with a HR of 1.96 (95% CI: 1.52 - 2.52), log-rank *P* = 1.1e-07. **(B)** Box plot comparing the expression levels of DLX2 between normal and tumor tissues, DLX2 is highly expressed in tumor tissues. The expression levels are represented as Log2 (TPM + 1), *** *P* < 0.001.

We performed a survival analysis of gastric cancer patients on the basis of DLX2 expression via Kaplan–Meier Plotter (http://kmplot.com). The results demonstrated that high DLX2 expression significantly reduced the survival rate of gastric cancer patients, with statistically significant differences observed in overall survival (OS), first progression (FP), and postprogression survival (PPS) (**Figure 1A**). These findings suggest that DLX2 plays a crucial role in the malignant progression of gastric cancer. To further investigate this, we extracted gastric adenocarcinoma data from the TCGA database and corresponding normal tissue data from the GTEx database via UCSC Xena. Our analysis revealed that DLX2 expression was significantly greater in tumor tissues than in normal tissues (**Figure 1B**, **Supplementary Figure S5**).

### DLX2 and its association with immunotherapy and drug sensitivity

**Figure 2:**
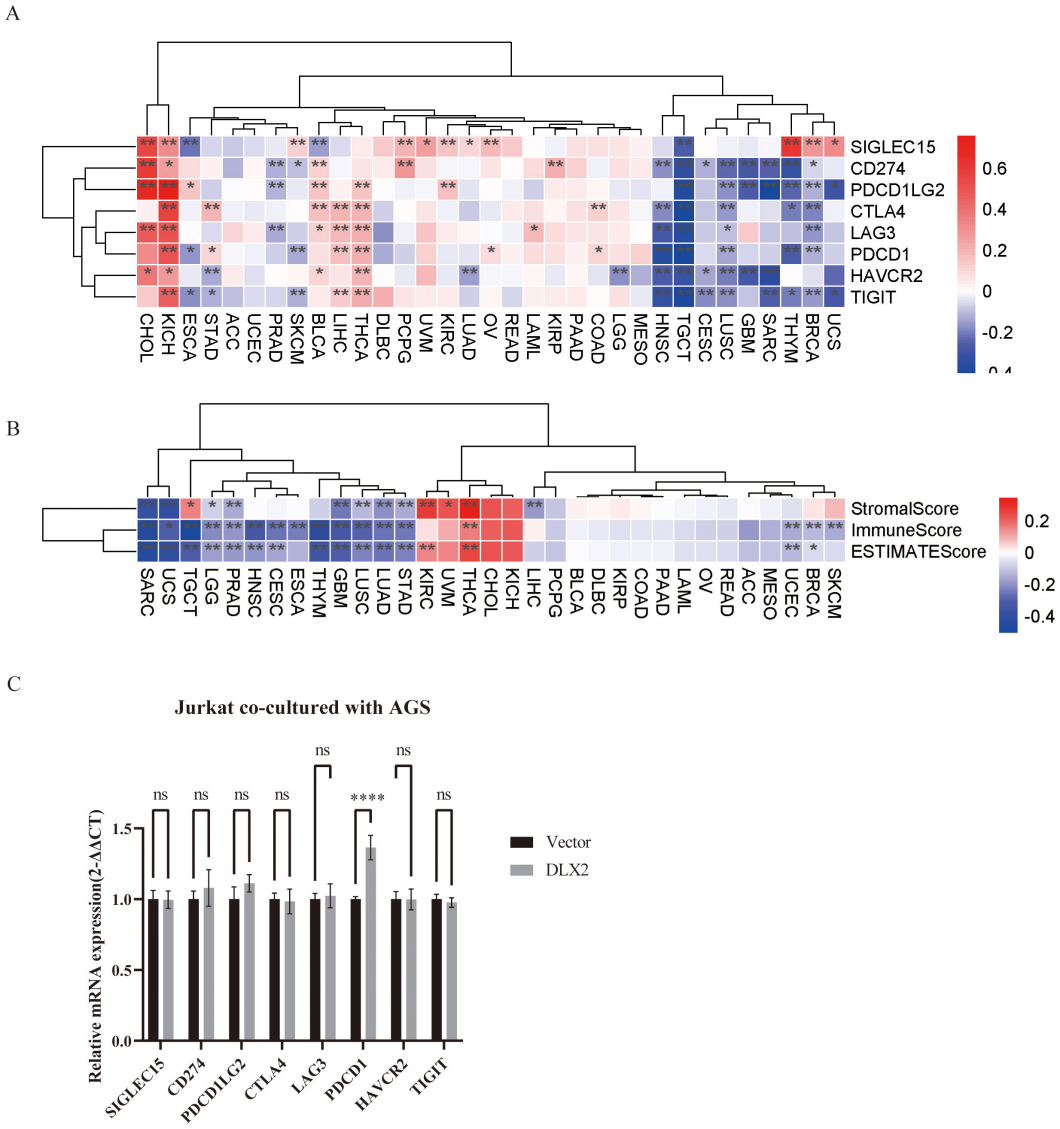
Pan-cancer analysis of the association between DLX2 and immune-related factors. **(A)** The heatmap illustrates the expression levels of specific immune checkpoint genes across various cancer types, along with their statistical significance. STAD (Stomach Adenocarcinoma) is associated with multiple immune checkpoint genes (CTLA4, PDCD1, HAVCR2, TIGIT). **(B)** Heatmap analysis of StromalScore, ImmuneScore, and ESTIMATEScore in high and low DLX2 expression groups across different cancer types. Among STAD, different DLX2 expression showed statistical differences in all three scores. **(C)** Bar graph showing the relative mRNA expression levels of immune checkpoint genes of Jurkat after co-culture with AGS cells (2-ΔΔCT method). ns P > 0.05, * *P* < 0.05, ** *P*<0.01, *** *P* < 0.001, **** *P* < 0.0001. NS indicates no significant difference, and ***p < 0.001 indicates a significant difference.

To explore whether DLX2 is associated with the immune microenvironment in gastric cancer, we obtained gene expression profiles from various tumors in the TCGA database and classified them into high and low DLX2 expression groups on the basis of the median expression level. We analyzed the correlation between DLX2 and several key immune checkpoints across different tumors via Pearson’s correlation analysis (**Figure 2A**). The research results indicated that there exists a significant positive correlation between DLX2 and CTLA4, PDCD1, HAVCR2 and TIGIT, which may contribute to the gastric cancer cells’ ability to evade immune system attacks. Additionally, in numerous other types of tumors, DLX2 is also closely associated with CD274 (PD-L1). Next, immune scoring analysis revealed a significant correlation between DLX2 expression and both immune and stromal scores in gastric cancer patients. The high DLX2 expression group presented decreased immune and stromal scores (**Figure 2B**), suggesting that DLX2 may play a crucial role in the tumor immune microenvironment by influencing immune evasion and stromal remodeling. Finally, we verified *in vitro* that DLX2 in gastric cancer cells can affect the expression of PDCD1 on the surface of T cells (**Figure 2C**).

To further explore the biological functions of the DLX2-related genes, we conducted functional enrichment analysis on the top positively and negatively correlated genes in gastric cancer (**Supplementary Figure S1**). Genes positively coexpressed with DLX2 were enriched primarily in biological processes such as forebrain neuron differentiation, neurogenesis, neural tube patterning, negative regulation of muscle tissue development, and regulation of muscle tissue development. In contrast, genes that were negatively coexpressed with DLX2 were predominantly associated with biological processes related to the humoral immune response, actomyosin contractile ring organization, antimicrobial peptide production, thrombin-activated receptor signaling, and negative regulation of NLRP3 inflammasome complex assembly. Additionally, we performed KEGG pathway analysis to identify genes co-expressed with DLX2 and the signaling pathways they are involved in. The results revealed that genes positively co-expressed with DLX2 are primarily involved in pathways such as salivary secretion, while those negatively co-expressed are involved in pathways like the TNF signaling pathway (**Supplementary Figure S1**). Additionally, gene set enrichment analysis (GSEA) based on DLX2 expression levels (**Supplementary Figure S2**) further revealed enrichment of multiple biological process gene sets in gastric cancer. Notably, gene sets related to digestion, DNA replication, and chromatid segregation were significantly enriched, suggesting that DLX2 may be associated with increased proliferative and metabolic activity in gastric cancer cells.

**Figure 3:**
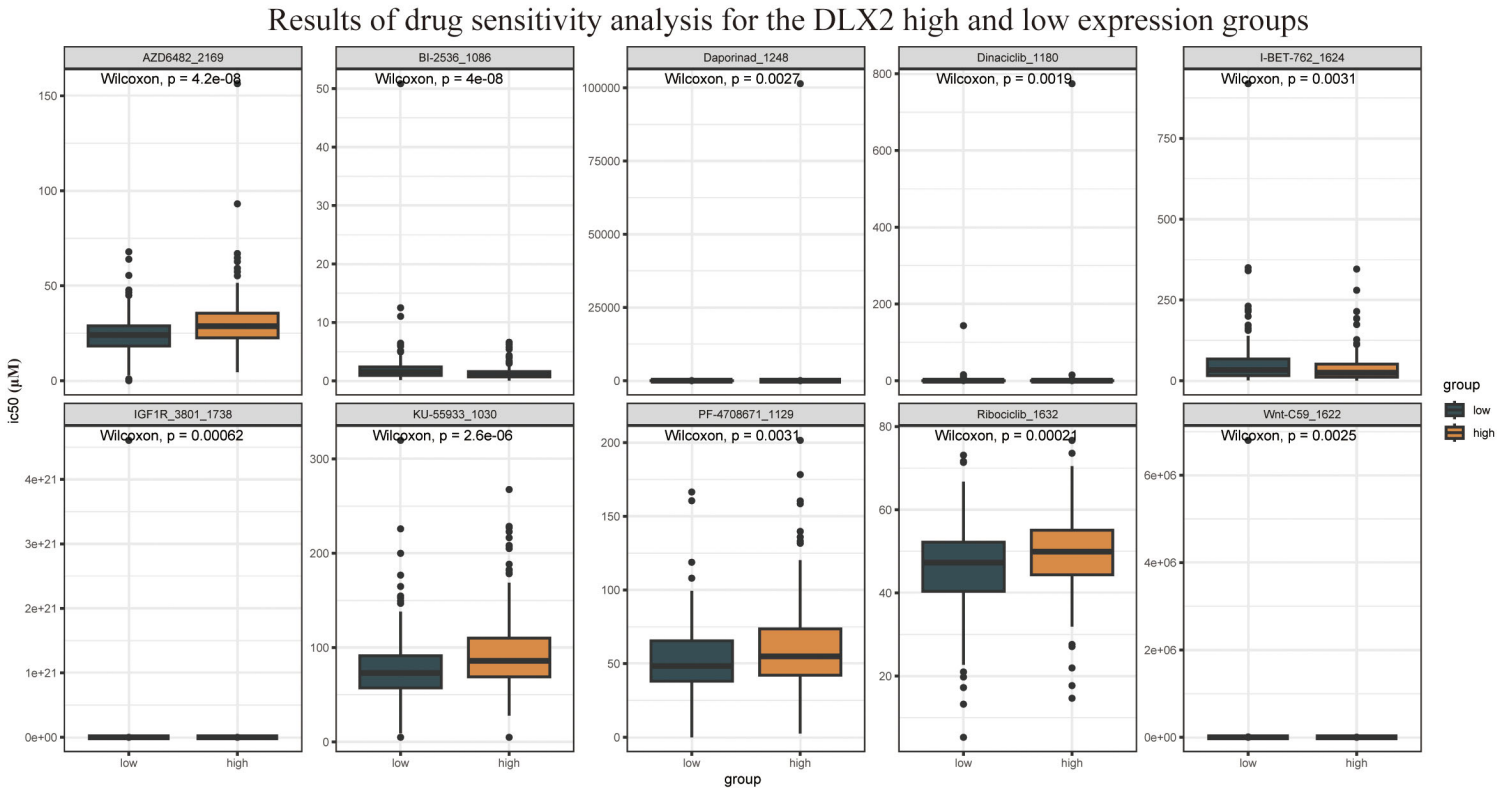
Drug sensitivity analysis results of DLX2 high and low expression groups.

Furthermore, drug sensitivity analysis revealed that DLX2 expression was correlated with sensitivity to multiple targeted therapeutic drugs (**Figure 3**), including AZD6482, dinaciclib, and ribociclib. Among these, AZD6482 is a PI3Kβ inhibitor, indicating that DLX2-related functions may be linked to the PI3K signaling pathway. These findings provide valuable insights for future investigations into the role of DLX2 in gastric cancer.

### DLX2 enhances gastric cancer cell proliferation

**Figure 4:**
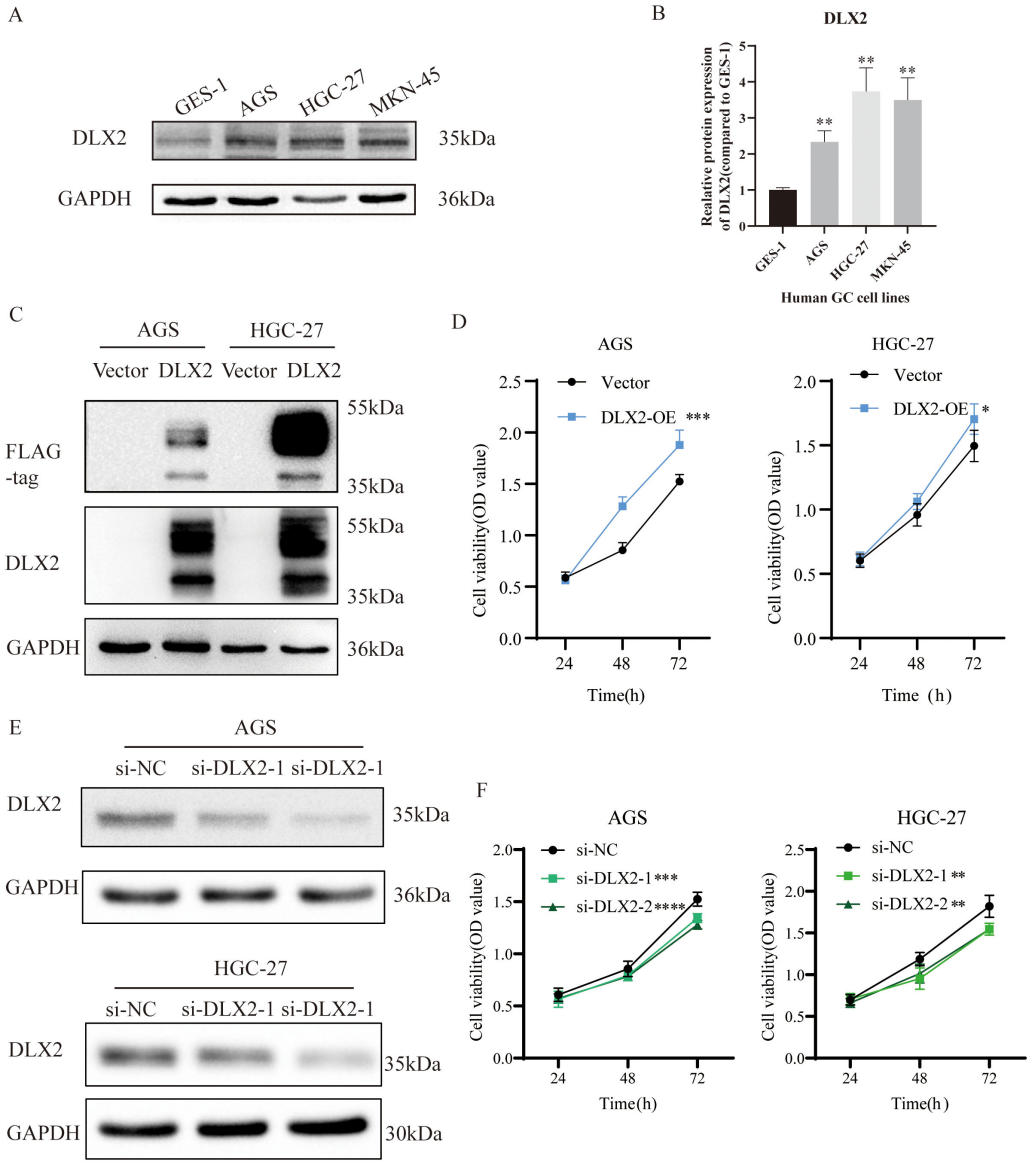
DLX2 promotes gastric cancer cell proliferation. **(A)** Protein expression levels of DLX2 in various gastric cancer cell lines. **(B)** Quantification of gray values from panel **(A)** C. Western blot validation of DLX2 overexpression in AGS/HGC-27 cells. **(D)** Effect of DLX2 overexpression on the proliferative capacity of AGS and HGC-27 cell lines assessed by CCK-8 assay. **(E)** Western blot analysis showing the efficiency of DLX2 gene silencing. GAPDH served as the loading control. **(F)** Effect of DLX2 gene silencing on the proliferative capacity of AGS and HGC-27 cell lines assessed by CCK-8 assay. * *P* < 0.05, ** *P*<0.01, *** *P* < 0.001, **** *P* < 0.0001.

To further investigate the role of DLX2 in gastric cancer progression, we examined its protein expression levels across various gastric cancer cell lines. DLX2 expression was significantly higher in gastric cancer cells than in normal gastric mucosal cells (GES-1) (**Figures 4A, B**). Moreover, expression levels varied among different gastric cancer cell lines. The elevated protein expression level of DLX2 in gastric cancer cells is consistent with the RNA sequencing data. On the basis of these findings, AGS and HGC-27 cell lines were selected for subsequent experiments. To explore the functional role of DLX2, we constructed a pcDNA3.1(+)-DLX2 overexpression plasmid for experimental studies. Following transient transfection and overexpression of DLX2 in gastric cancer cell lines, Western blot analysis confirmed successful overexpression (**Figure 4C**). The multiple bands detected by the anti-Flag antibody in the Western blotting of transiently overexpressed exogenous DLX2 are likely due to post-translational modifications or oligomerization of DLX2. It is worth noting that the predicted molecular weight band (35 kDa) is also present, confirming the existence of the normal monomer or unmodified state of DLX2. To assess the functional impact of DLX2 on gastric cancer cells, we performed CCK-8 proliferation assays. The results revealed that after 72 hours of DLX2 overexpression, the number of AGS and HGC-27 cells in the experimental group was significantly greater than that in the control group (pcDNA3.1(+)) (**Figure 4D**), indicating a protumorigenic role of DLX2 in gastric cancer. Conversely, transient transfection of siRNA to silence DLX2 expression effectively reduced its protein level, as confirmed by Western blot analysis of AGS and HGC-27 cells (**Figure 4E**). A CCK-8 assay further revealed that DLX2 knockdown suppressed gastric cancer cell proliferation, supporting its oncogenic role in gastric cancer (**Figure 4F**).

### DLX2 promotes gastric cancer cell migration and invasion

**Figure 5:**
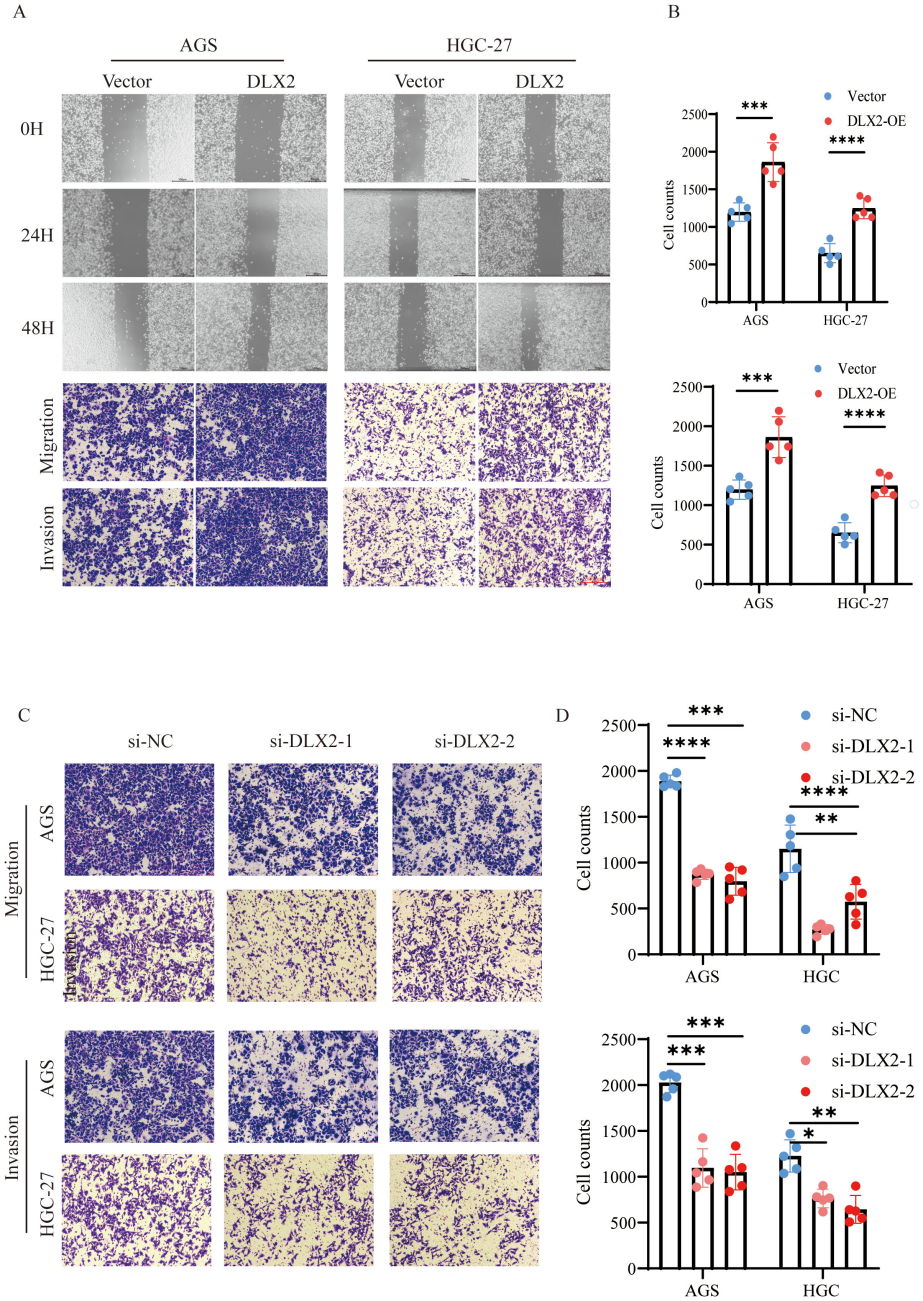
DLX2 promotes gastric cancer cell migration and invasion. **(A)** Migratory capacity of AGS and HGC-27 cells in the control group (Vector) and DLX2 overexpression group (DLX2-OE) was assessed by wound healing assay. Representative images of cell migration at 0, 24, and 48 hours are shown. **(B)** Transwell migration and invasion assays of AGS and HGC-27 cells in both groups were analyzed by crystal violet staining. **(C)** Migration assay of AGS and HGC-27 cells following DLX2 silencing. **(D)** Invasion assay of AGS and HGC-27 cells following DLX2 silencing. Data are presented as mean ± SD; (n = 5). Statistical significance was determined using two-tailed Student’s (*t*)-test: * *P* < 0.05, ** *P*<0.01, *** *P* < 0.001, **** *P* < 0.0001. Scale bar: 100 µm.

To evaluate the effect of DLX2 on gastric cancer cell migration and invasion, we performed wound healing and Transwell assays. A wound healing assay demonstrated that DLX2 overexpression significantly accelerated cell migration (**Figure 5A**). Similarly, the Transwell assay results revealed that DLX2 overexpression markedly enhanced the migration and invasion of gastric cancer cells (**Figure 5B**). In contrast, siRNA-mediated knockdown of DLX2 significantly reduced the migratory and invasive abilities of AGS and HGC-27 cells (**Figures 5C, D**). These findings suggest that DLX2 enhances gastric cancer cell migration and invasion, further supporting its oncogenic role.

### *In vivo* validation of the oncogenic role of DLX2 in gastric cancer

**Figure 6:**
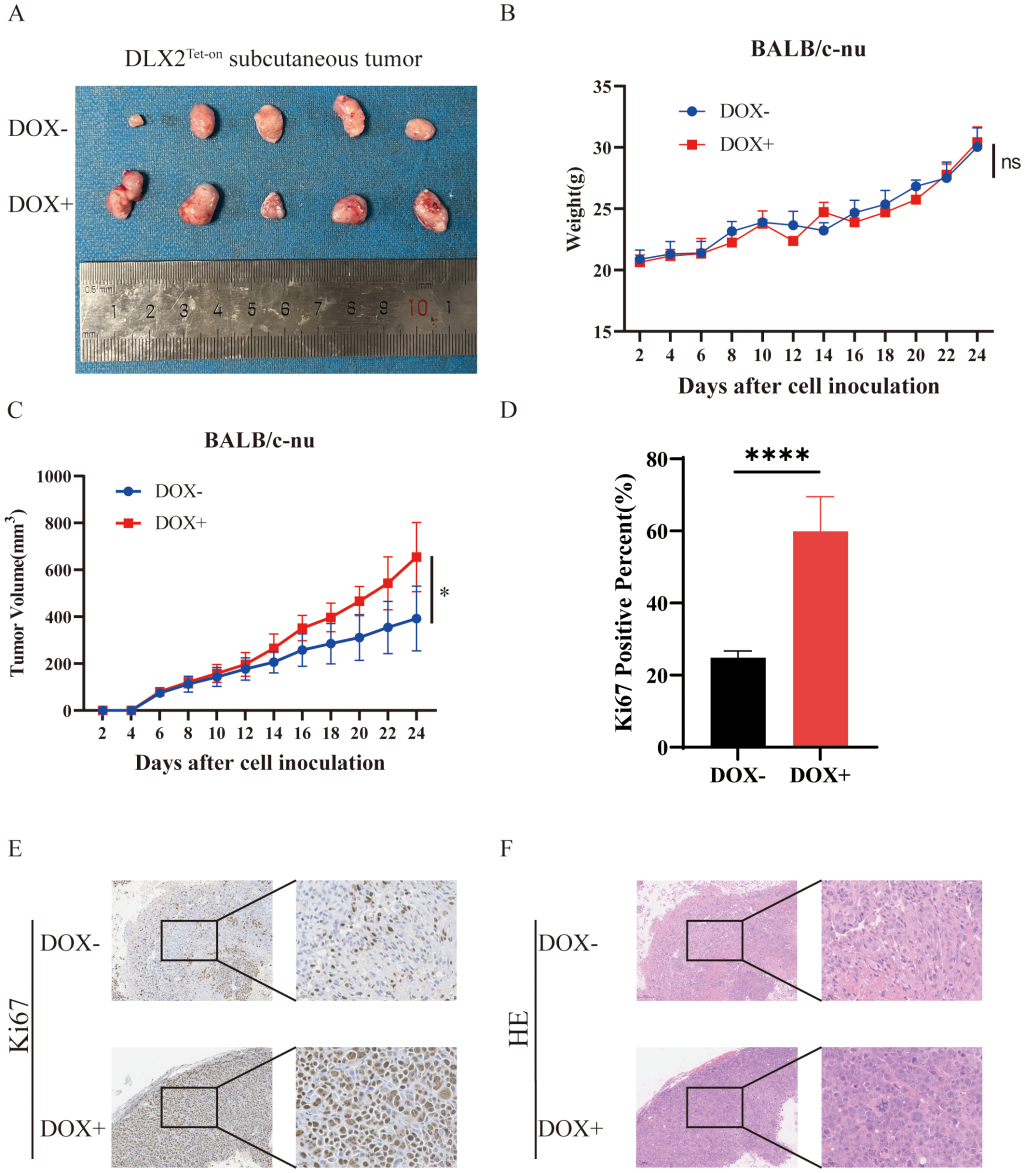
DLX2 promotes tumor growth and progression *in vivo*. **(A)** Representative images of tumor tissues. Differences in tumor volume between mice under DOX− (untreated) and DOX+ (doxycycline-induced) conditions are shown. Larger tumor volumes were observed in the DOX+ group. **(B)** Body weight changes of mice over the experimental timeline. **(C)** Tumor volume growth curves. Tumor volumes in DOX− and DOX+ groups were measured at indicated time points. The DOX+ group exhibited accelerated tumor growth. **(D, E)** Representative Ki-67 IHC staining of tumor tissues. **(F)** Hematoxylin and eosin (H&E) staining of tumor tissues. ns P > 0.05, * P < 0.05, **** P < 0.0001.

To validate the biological function of DLX2 in gastric cancer, we conducted *in vivo* experiments. A stable DLX2-overexpressing gastric cancer cell line was established via the Tet-on system and subcutaneously injected into BALB/c-nu nude mice. The mice were divided into a doxycycline (Dox, 1 mg/ml) oral induction group and a control group, and tumor growth was monitored every other day. The results revealed that the tumors in the Dox-induced DLX2 overexpression group were significantly larger than those in the control group (**Figures 6A–C**) (*P < 0.05*). These findings indicate that DLX2 expression enhances tumor growth in gastric cancer. Histological analysis via hematoxylin–eosin (HE) staining (**Figure 6E**) and Ki-67 immunohistochemistry (IHC) staining (**Figures 6D, F**) further supported these findings. High Ki-67 expression in the Dox+ experimental group indicated increased tumor cell proliferation. These *in vivo* results validate the oncogenic role of DLX2 in gastric cancer.

### DLX2 promotes the EMT process in gastric cancer cells

**Figure 7:**
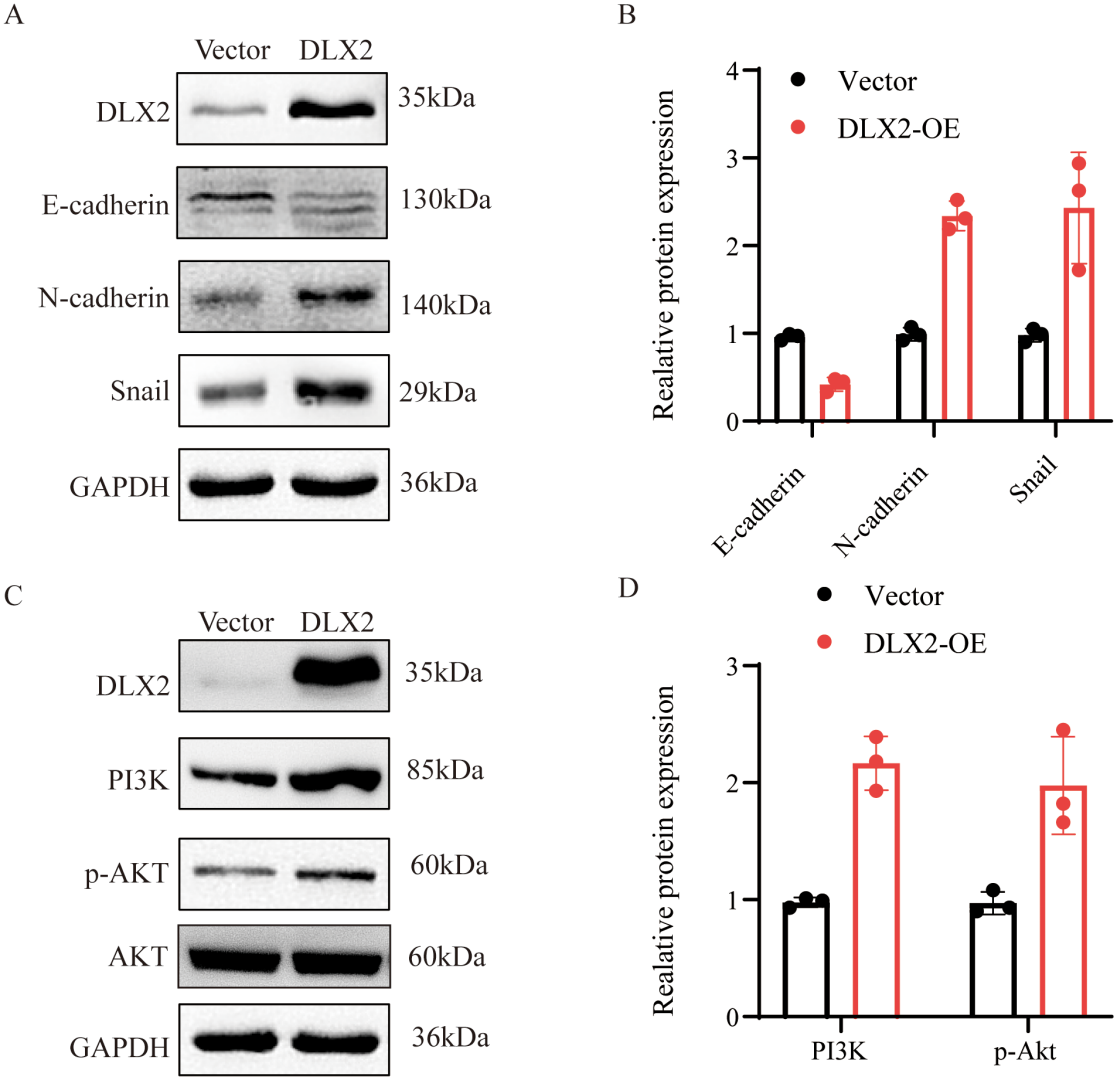
Effect of DLX2 expression manipulation on epithelial-mesenchymal transition (EMT) protein levels in gastric cancer cells. **(A, B)** The expression of E-cadherin, N-cadherin, and Snail protein in AGS cells of the DLX2 group and Vector group. **(C, D)** The expression of PI3K and p-AKT protein in AGS cells of the DLX2 group and Vector group. The relative expression levels of individual molecules were determined by normalizing their levels with that of GAPDH and then compared to that of vector, which was set as 1.

This study demonstrated the role of DLX2 in promoting GC proliferation, migration, and invasion through *in vitro* functional assays and *in vivo* xenograft tumor models. EMT is a critical process in tumor progression that promotes cell migration and invasion. EMT is characterized by the downregulation of epithelial markers (e.g., E-cadherin) and the upregulation of mesenchymal markers (e.g., N-cadherin), facilitating tumor metastasis. In AGS cells, DLX2 overexpression led to a decrease in E-cadherin expression, whereas N-cadherin and Snail expression significantly increased (**Figures 7A, B**). These results suggest that DLX2 promotes EMT, thereby enhancing gastric cancer malignancy.

### DLX2 activates the PI3K/AKT signaling pathway in gastric cancer

Drug sensitivity analysis revealed a correlation between DLX2 expression and sensitivity to AZD6482, a PI3Kβ inhibitor, suggesting a potential link between DLX2 and the PI3K signaling pathway. Since the PI3K/AKT pathway plays a crucial role in tumor EMT, we further examined this relationship. Western blot analysis following DLX2 overexpression revealed a significant increase in PI3K expression (**Figures 7C, D**). Additionally, phosphorylated AKT (p-AKT) levels were markedly elevated, indicating activation of the PI3K/AKT pathway. These findings suggest that DLX2 promotes gastric cancer EMT, migration, and invasion by activating the PI3K/AKT signaling pathway, thereby increasing tumor progression. Furthermore, we confirmed that the impact of DLX2 on the malignancy of gastric cancer can be reversed by PI3K/AKT inhibitors, indicating that DLX2 promotes the progression of gastric cancer malignancy through the PI3K/AKT pathway (**Supplementary Figure S6**).

## Discussion

The mechanisms underlying tumor initiation and progression remain unclear. The incidence and mortality rates of GC continue to be high, particularly in East Asian countries, which poses a significant therapeutic challenge that has yet to be overcome. In this study, we demonstrated that DLX2 serves as a critical oncogenic driver in GC by promoting EMT and malignant progression through activation of the PI3K/AKT signaling pathway. Our findings revealed that elevated DLX2 expression is correlated with poor prognosis and enhanced tumor proliferation, migration, invasion, and immune evasion, positioning DLX2 as both a prognostic biomarker and a potential therapeutic target in GC. These results are consistent with and extend previous studies implicating DLX2 in cancer progression while providing novel insights into its mechanistic roles in GC biology.

DLX2 is a transcription factor of the DLX family, which consists of DLX1, DLX2, DLX3, DLX4, and DLX5. Our previous analysis identified DLX4 and DLX2 as biomarkers linked to histological grade and prognosis in gastric cancer (GC) (30). DLX4 has been implicated in shaping an immunosuppressive microenvironment by regulating cell cycle, EMT, glycolysis, and inflammatory pathways, and its high expression correlates with poor survival in GC through the PD-L1/GATA1 axis (31). DLX2 has also emerged as a poor prognostic factor in colorectal cancer (32) and liver cancer, where it promotes sorafenib resistance via EMT activation and ERK signaling (32). DLX5 exerts oncogenic effects in osteosarcoma through NOTCH1 activation (19), and given that the Notch pathway drives TGF-β–induced EMT (33), DLX2 may contribute to EMT through similar mechanisms. In GC, DLX2 is markedly upregulated in tumor versus normal tissues (34). Consistent with this, our study confirmed its high expression in GC and demonstrated that elevated DLX2 predicts unfavorable survival outcomes. These findings highlight DLX2 as a potential oncogenic driver and independent prognostic marker in GC, with implications for risk stratification and personalized therapy.

The tumor microenvironment (TME) comprises various immune cell types, cancer-associated fibroblasts, endothelial cells, pericytes, and other tissue-resident cell types, all of which play crucial roles in cancer pathogenesis (35). Immune checkpoints are a group of molecules expressed on immune cells that regulate the degree of immune activation. Aberrant expression and dysfunction of immune checkpoint molecules are key contributors to cancer immune evasion (36). Tumor cells can exploit immune checkpoints, such as PD-1, to evade immune surveillance, allowing them to survive and proliferate (37).

The positive association between DLX2 and immune checkpoints, such as CD274 (PD-L1) and SIGLEC15, suggests a dual mechanism: DLX2 may facilitate immune evasion by upregulating immunosuppressive molecules while fostering stromal interactions that promote metastasis. Similar immunomodulatory roles of DLX2 have been reported in lung squamous cell carcinoma, and DLX2 was found to be associated with the IPS of PD-1/PD-L1 blockade as well as CTLA-4 combined with PD-1/PD-L1 blockade (27). Functional enrichment analysis further revealed that DLX2-associated pathways include neuronal differentiation, muscle development, and immune regulation, highlighting its pleiotropic influence on GC progression. These findings suggest that targeting DLX2 could disrupt both tumor-intrinsic and microenvironmental mechanisms of immune suppression, providing a rationale for combining DLX2 inhibitors with immunotherapies.

This study validated the role of DLX2 in promoting GC proliferation, migration, and invasion through both *in vitro* functional assays and *in vivo* xenograft tumor models. Additionally, we explored the underlying mechanisms and reported that DLX2 overexpression influences the expression of EMT-related factors, such as E-cadherin, N-cadherin, and Snail, thereby promoting the EMT process.

Moreover, DLX2 drives GC malignancy by activating the PI3K/AKT pathway, a well-established regulator of EMT and tumor aggressiveness. Overexpression of DLX2 in GC cells led to increased PI3K and phosphorylated AKT levels, while DLX2 knockdown suppressed these effects. This finding is consistent with the drug sensitivity data indicating that DLX2 expression is associated with responsiveness to PI3Kβ inhibitors such as AZD6482, further supporting its reliance on PI3K/AKT signaling. Activation of this pathway by DLX2 likely underlies its ability to induce EMT, as evidenced by E-cadherin downregulation and N-cadherin/Snail upregulation—hallmarks of metastatic progression (38). The PI3K/AKT axis is known to promote EMT and chemoresistance in GC by modulating downstream effectors such as mTOR and GSK-3β (39). These interconnected mechanisms position DLX2 as a central regulator of GC progression, orchestrating both cellular plasticity and immune escape.

Therapeutic targeting of DLX2 holds promise but requires careful consideration. Preclinical studies in hepatocellular carcinoma have demonstrated that DLX2 silencing suppresses tumor proliferation and metastasis (40). In GC, combining DLX2 inhibitors with PI3K/AKT pathway blockers or immune checkpoint inhibitors could yield synergistic effects. However, the precise mechanisms linking DLX2 to these pathways remain unclear, and further studies are needed to identify its direct targets. Additionally, larger patient cohorts are required to validate these findings, and the lack of specific DLX2 inhibitors remains a challenge for clinical application, highlighting the need for novel combination therapies. Future research should explore the role of DLX2 in therapy-resistant niches and its crosstalk with stromal components, such as cancer-associated fibroblasts and immune cells, to fully elucidate its impact on the TME.

In conclusion, this study establishes DLX2 as a crucial regulator of GC malignancy, driving EMT and PI3K/AKT activation to fuel aggressive tumor phenotypes. These findings enhance our understanding of GC pathogenesis and provide a compelling rationale for targeting DLX2 in precision oncology. Further investigations into its interplay with the TME and therapeutic vulnerabilities will be critical for translating these insights into clinical applications.

## Data Availability

The original contributions presented in the study are included in the article/**Supplementary Material**. Further inquiries can be directed to the corresponding author.

## Supplementary material

Supplementary Figure 1 Functional enrichment analysis of DLX2-associated genes. **(A)** Correlation analysis between DLX2 gene expression and patient survival rates (Kaplan-Meier survival curves). **(B)** Gene Ontology (GO) functional enrichment analysis of DLX2 co-expressed genes. **(C)** Heatmap analysis of DLX2 co-expressed genes. **(D, E)** KEGG pathway analysis of genes positively and negatively co-expressed with DLX2. Gene expression levels are represented by a color gradient from blue (low expression) to red (high expression).

Supplementary Figure 2 Gene set enrichment analysis (GSEA) in stomach adenocarcinoma (STAD). **(A)** GO analysis revealed that DLX2 is associated with digestion, DNA-templated DNA replication, DNA geometric change, and other related processes. **(B)** Kyoto Encyclopedia of Genes and Genomes (KEGG) pathway analysis showed that DLX2 is related to autoimmune thyroid disease, cell cycle, intestinal immune network for IgA production, and other pathways.

Supplementary Figure 3 Immune infiltration analysis of DLX2 gene expression in gastric cancer based on TCGA-STAD database. Samples were divided into high and low expression groups according to the median DLX2 expression level. Eight computational algorithms from the IOBR package, including CIBERSORT, EPIC, ssGSEA, MCP_counter, Quanti-seq, TIMER, xCell, and Estimate, were used to assess the infiltration levels of various immune cell types. The figure highlights the distribution differences of different immune cell types between the high and low DLX2 expression groups and their correlations with clinical characteristics such as gender, stage, diagnosis, and survival status.

Supplementary Figure 4 The correlation between DLX2 expression and immune cell infiltration in gastric cancer. The lollipop plot illustrates the relationship between DLX2 levels and various immune cell types based on multiple computational algorithms. Positive and negative associations are indicated by the direction of the lollipop stems, with the length representing the strength of the correlation. Statistical significance is marked accordingly, highlighting the potential role of DLX2 in modulating the tumor immune microenvironment in gastric cancer.

Supplementary Figure 5 Expression of DLX2 in GSE54129. Box plot comparing the expression levels of DLX2 between normal and tumor tissues, DLX2 is highly expressed in tumor tissues.

Supplementary Figure 6 DLX2 promotes gastric cancer through PI3K/AKT pathway. **(A)** The effect of DLX2 overexpression or co-treatment with AZD6482 on cell viability in AGS and HGC-27 cell lines. Overexpression of DLX2 significantly increased cell viability, which was inhibited by AZD6482 treatment (*p < 0.05, **p < 0.01). **(B)** Western blot analysis showing protein expression levels of DLX2, PI3K, p-AKT, and AKT. GAPDH was used as a loading control. **(C)** Transwell assays demonstrating the impact of DLX2 overexpression on the migration and invasion capabilities of AGS and HGC-27 cells.
