# Supplementary figures and images for: DLX2 promotes gastric cancer epithelial– mesenchymal transition and malignant progression through the PI3K/AKT signaling pathway
Source: Front Oncol. 2025 Oct 30;15:1669890. doi: 10.3389/fonc.2025.1669890 (PMC12612836; doi:10.3389/fonc.2025.1669890)

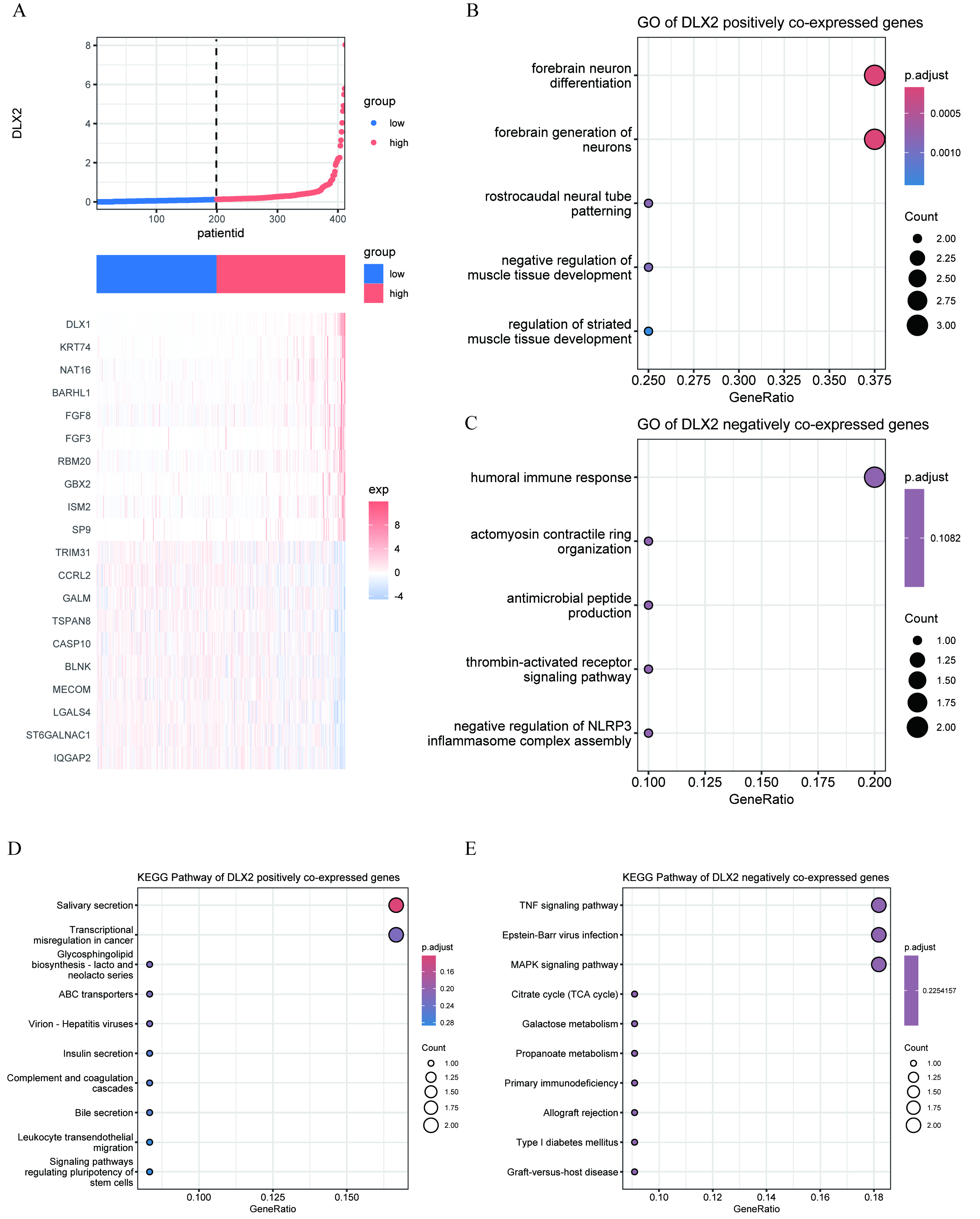

Supplement: Supplementary Figure 1 — Functional enrichment analysis of DLX2-associated genes. (A) Correlation analysis between DLX2 gene expression and patient survival rates (Kaplan-Meier survival curves). (B) Gene Ontology (GO) functional enrichment analysis of DLX2 co-expressed genes. (C) Heatmap analysis of DLX2 co-expressed genes. (D, E) KEGG pathway analysis of genes positively and negatively co-expressed with DLX2. Gene expression levels are represented by a color gradient from blue (low expression) to red (high expression). [file Image1.tif]

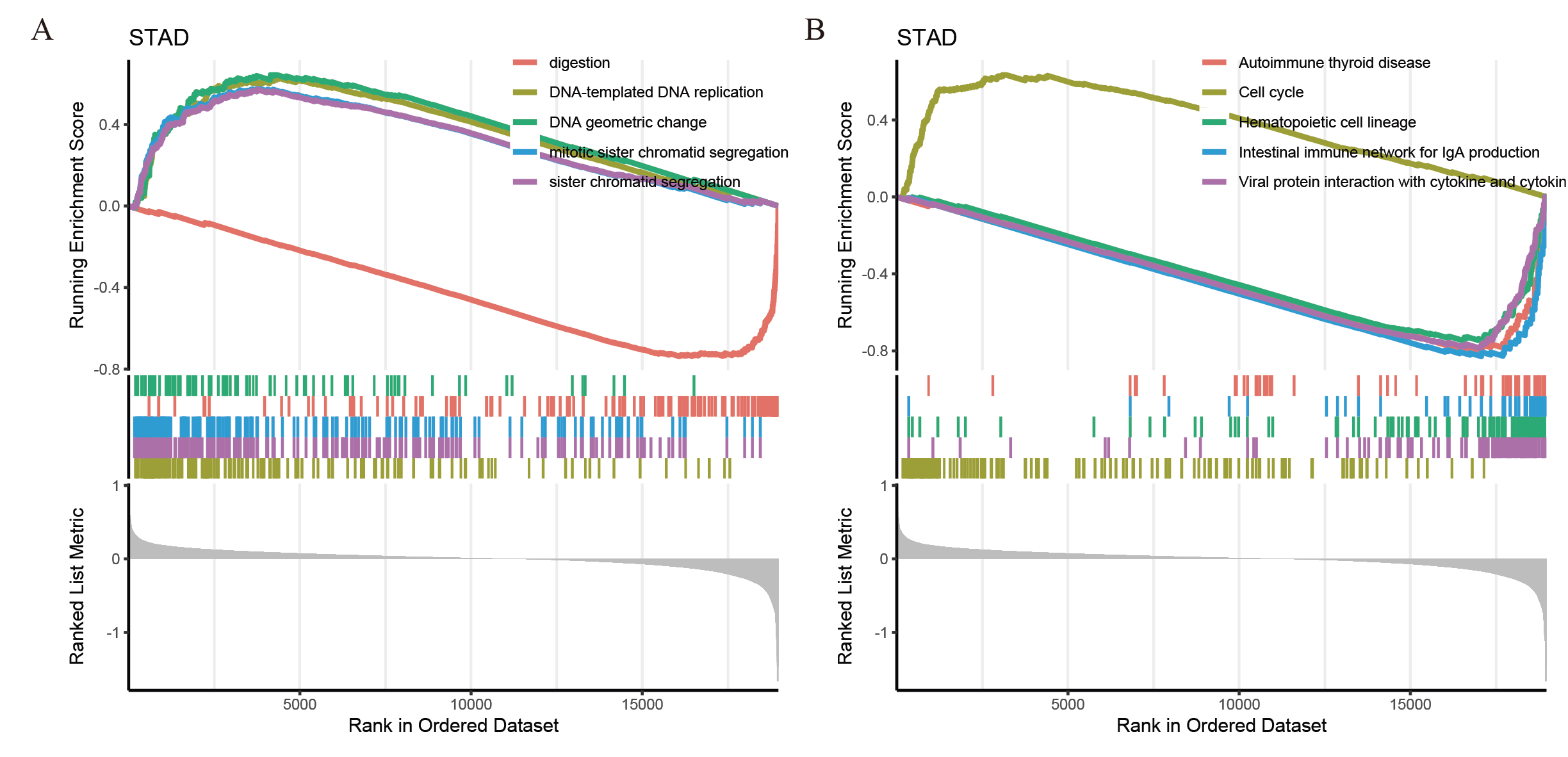

Supplement: Supplementary Figure 2 — Gene set enrichment analysis (GSEA) in stomach adenocarcinoma (STAD). (A) GO analysis revealed that DLX2 is associated with digestion, DNA-templated DNA replication, DNA geometric change, and other related processes. (B) Kyoto Encyclopedia of Genes and Genomes (KEGG) pathway analysis showed that DLX2 is related to autoimmune thyroid disease, cell cycle, intestinal immune network for IgA production, and other pathways. [file Image2.tif]

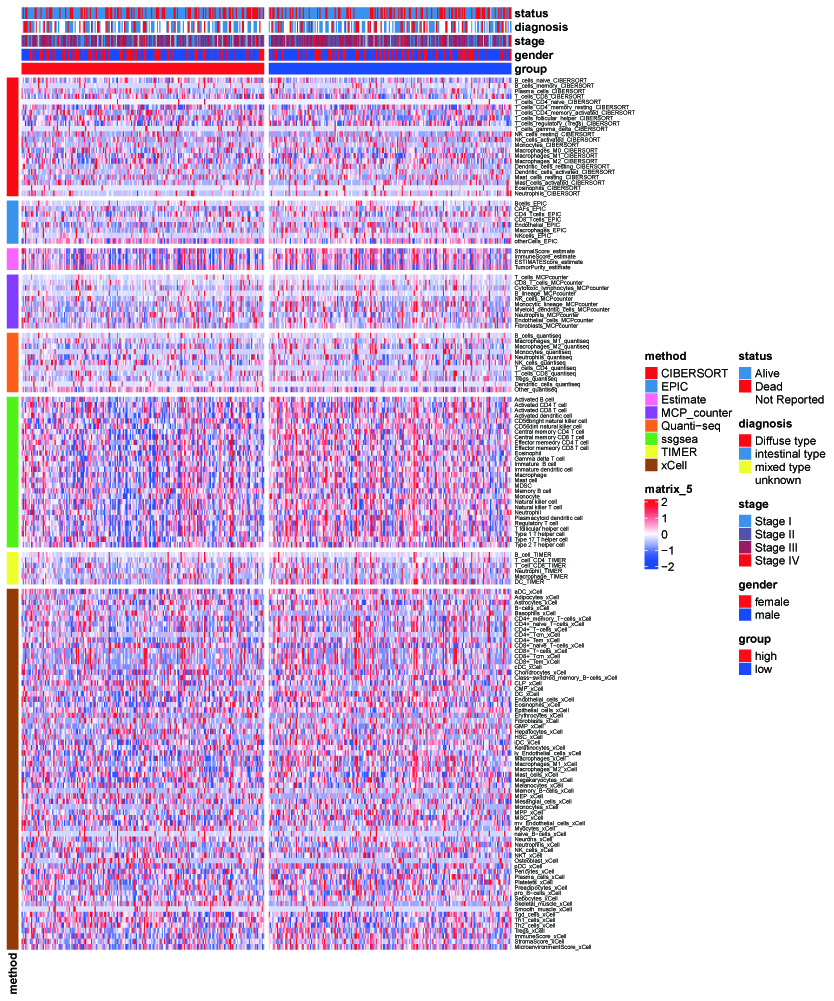

Supplement: Supplementary Figure 3 — Immune infiltration analysis of DLX2 gene expression in gastric cancer based on TCGA-STAD database. Samples were divided into high and low expression groups according to the median DLX2 expression level. Eight computational algorithms from the IOBR package, including CIBERSORT, EPIC, ssGSEA, MCP_counter, Quanti-seq, TIMER, xCell, and Estimate, were used to assess the infiltration levels of various immune cell types. The figure highlights the distribution differences of different immune cell types between the high and low DLX2 expression groups and their correlations with clinical characteristics such as gender, stage, diagnosis, and survival status. [file Image3.tif]

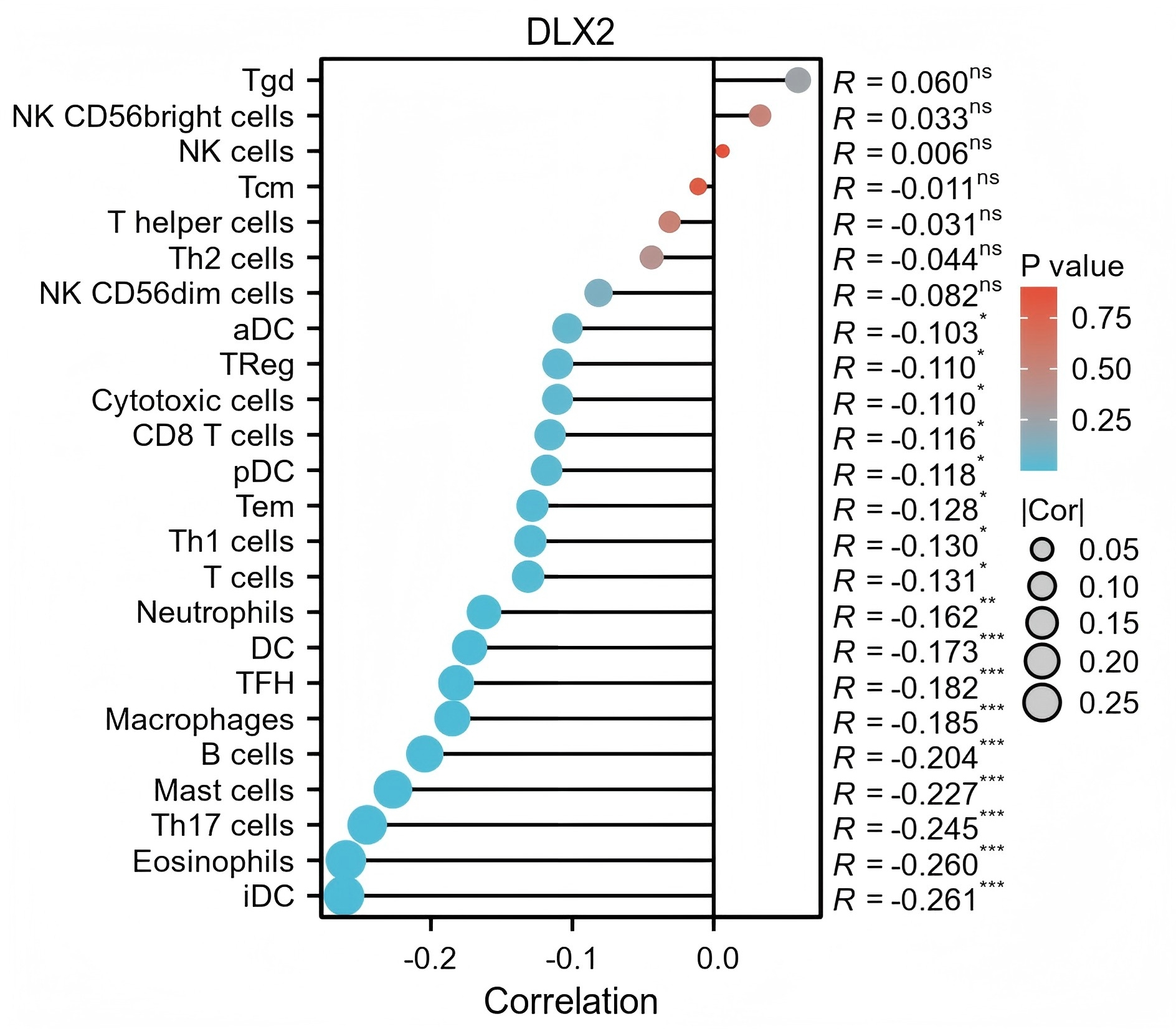

Supplement: Supplementary Figure 4 — The correlation between DLX2 expression and immune cell infiltration in gastric cancer. The lollipop plot illustrates the relationship between DLX2 levels and various immune cell types based on multiple computational algorithms. Positive and negative associations are indicated by the direction of the lollipop stems, with the length representing the strength of the correlation. Statistical significance is marked accordingly, highlighting the potential role of DLX2 in modulating the tumor immune microenvironment in gastric cancer. [file Image4.jpeg]

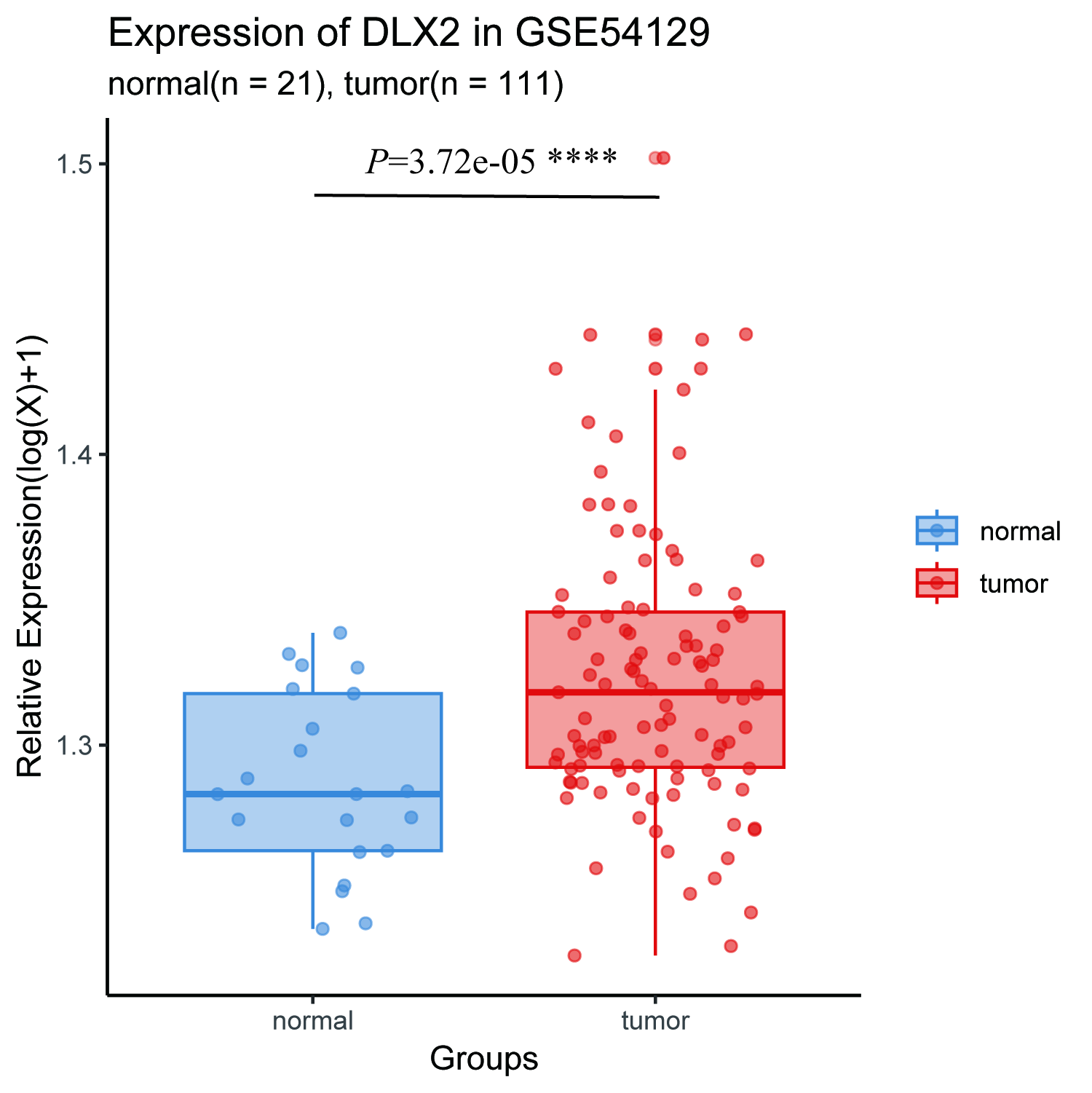

Supplement: Supplementary Figure 5 — Expression of DLX2 in GSE54129. Box plot comparing the expression levels of DLX2 between normal and tumor tissues, DLX2 is highly expressed in tumor tissues. [file Image5.tif]

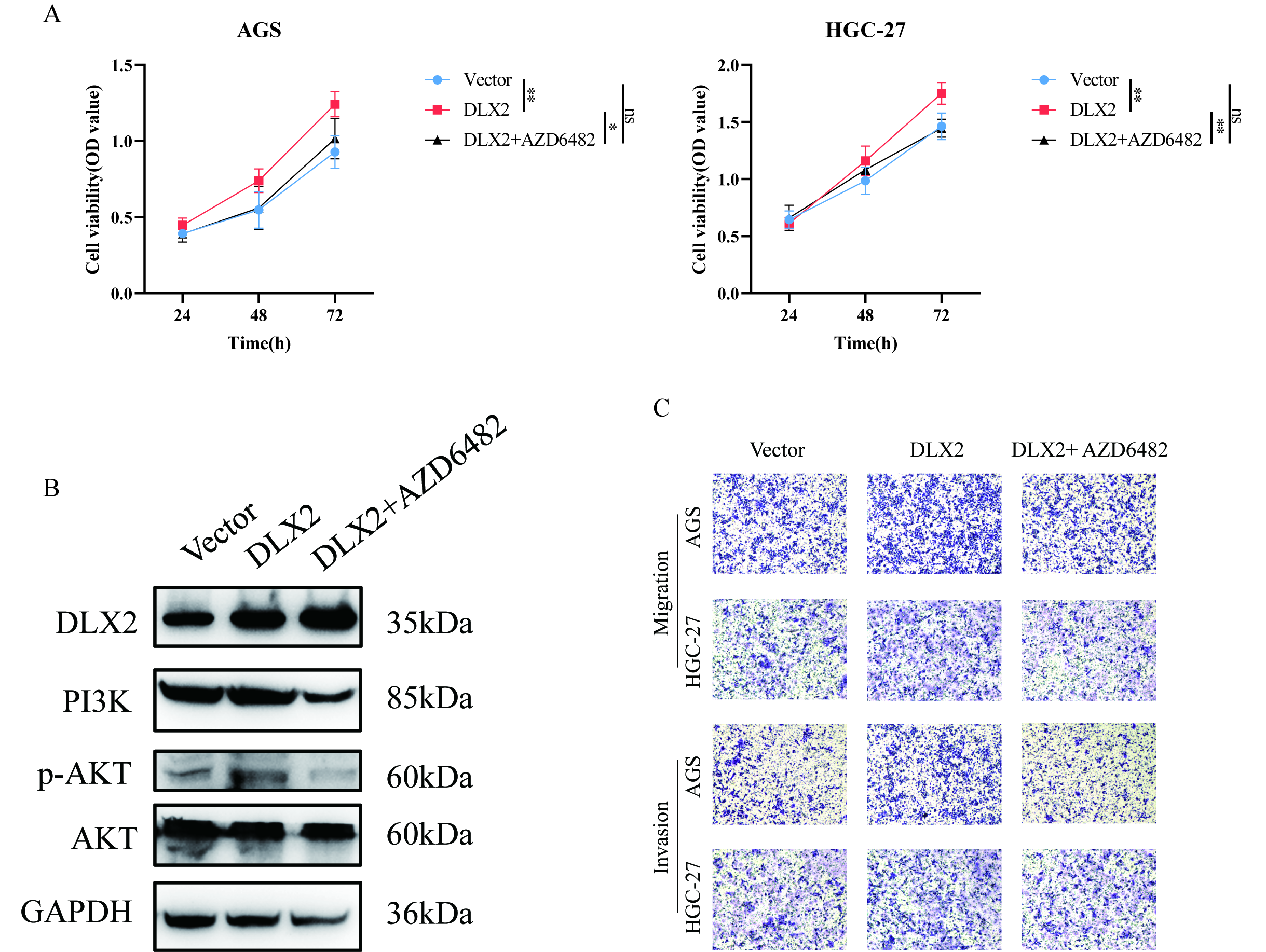

Supplement: Supplementary Figure 6 — DLX2 promotes gastric cancer through PI3K/AKT pathway. (A) The effect of DLX2 overexpression or co-treatment with AZD6482 on cell viability in AGS and HGC-27 cell lines. Overexpression of DLX2 significantly increased cell viability, which was inhibited by AZD6482 treatment (*p < 0.05, **p < 0.01). (B) Western blot analysis showing protein expression levels of DLX2, PI3K, p-AKT, and AKT. GAPDH was used as a loading control. (C) Transwell assays demonstrating the impact of DLX2 overexpression on the migration and invasion capabilities of AGS and HGC-27 cells. [file Image6.tif]
